# Standardized post-resuscitation damage assessment of two mechanical chest compression devices: a prospective randomized large animal trial

**DOI:** 10.1186/s13049-021-00892-4

**Published:** 2021-06-05

**Authors:** Robert Ruemmler, Jakob Stein, Bastian Duenges, Miriam Renz, Erik Kristoffer Hartmann

**Affiliations:** Department of Anaesthesiology, Medical Centre of the Johannes Gutenberg-University, Langenbeckstrasse 1, 55131 Mainz, Germany

**Keywords:** Mechanical chest compression devices, Post-resuscitation injuries, MIGET, Porcine, LUCAS, Corpuls

## Abstract

**Background:**

Mechanical chest compression devices are accepted alternatives for cardiopulmonary resuscitation (CPR) under specific circumstances. Current devices lack prospective and comparative data on their specific cardiovascular effects and potential for severe thoracic injuries.

**Objectives:**

To compare CPR effectiveness and thoracic injuries of two mechanical chest compression devices in pigs.

**Study design:**

Prospective randomised trial.

**Animals:**

Eighteen male German landrace pigs.

**Methods:**

Ventricular fibrillation was induced in anaesthetised and instrumented pigs and the animals were randomised into two intervention groups. Mechanical CPR was initiated by means of LUCAS™ 2 (mCCD1) or Corpuls™ cpr (mCCD2) device. Advanced life support was applied for a maximum of 10 cycles and animals achieving ROSC were monitored for 8 h. Ventilation/perfusion measurements were performed and blood gas analyses were taken. Thoracic injuries were assessed via a standardised damage score.

**Results:**

Five animals of the mCCD1 group and one animal of the mCCD2 group achieved ROSC (*p* = 0.048). Only the mCCD1 animals survived until the end of the monitoring period (*p* < 0.01). MCCD1 animals showed less pulmonary shunt (*p* = 0.025) and higher normal V/Q (*p* = 0.017) during CPR. MCCD2 animals showed significantly more severe thoracic injuries (*p* = 0.046).

**Conclusion:**

The LUCAS 2 device shows superior resuscitation outcomes and less thoracic injuries compared to Corpuls cpr when used for experimental CPR in juvenile pigs. Researchers should be aware that different mCCDs for experimental studies may significantly influence the respective outcome of resuscitation studies and affect comparability of different trials. Controlled human and animal CPR studies and a standardised post-resuscitation injury evaluation could help to confirm potential hazards.

**Trial registration:**

Trial approval number: G16–1-042-E4.

**Supplementary Information:**

The online version contains supplementary material available at 10.1186/s13049-021-00892-4.

## Background

Mechanical chest compression devices (mCCDs) have been increasingly implemented into clinical and out-of-hospital emergency care over the past 2 decades [1, 2]. The predominantly electric devices can be able to facilitate continuous and high-quality cardiopulmonary resuscitation (CPR) and provide adequate organ perfusion under cardiac arrest even during prolonged patient transport and when confronted with limited provider resources [3, 4]. While there is no sufficient evidence for superior patient outcomes after mCCD use compared to standard manual CPR [5–7], the European Resuscitation Council (ERC) explicitly accepts their use in their most recent guidelines as an alternative therapeutic method in adverse resuscitation scenarios [8]. However, despite the fact that several different devices and mechanical strategies are available, randomised clinical trials are scarce and are the basis of a weak level of treatment recommendations. The majority of mCCD trials have been performed using the Lund University cardiac arrest system (LUCAS™) and its 2nd and 3rd generation successors [9, 7], making it the most reliable source for comparative data. Since the last ERC guideline update in 2015, another piston-mounted compression device, the Corpuls™ cpr has been approved for medical use, but currently lacks detailed prospective assessment [10–12].

In translational and basic CPR research, large animal models play an extraordinary role for assessment of new treatment strategies or devices, as controlled clinical examinations in patients receiving CPR are inevitably limited by ethical reservations. This highlights the need to identify confounding factors that may influence the respective outcome of the study and complicate comparability.

We hypothesised that experimental CPR in juvenile pigs is not particularly influenced by different mCCDs. We used an established, prospective large animal resuscitation model to compare CPR efficiency, return of spontaneous circulation (ROSC) rates and thoracic injuries after prolonged resuscitation with two commercially available mCCDs. Secondly, we propose to develop a standardised post-resuscitation damage assessment tool in the process.

## Methods

### Anaesthesia/instrumentation

After approval of the experimental protocol by the State and Institutional Animal Care Committee Rhineland Palatine (approval no. G16–1-042-E4), 18 male German landrace pigs (age range 16–20 weeks, weighing 30–35 kg) were acquired from a local farm after being screened by the breeder for any obvious medical conditions or diseases according to the German Animal Care Regulations. The animals were given pre-transport sedation via an intramuscular injection of azaperone (2 mg kg^− 1^) and ketamine (4 mg kg^− 1^) and were secured in a large box with hay bedding, in which they were transported to our facility (~ 30 min). After the pigs were in the Large Animal Research Facility, anaesthesia was induced via an intravenous catheter placed in the lateral/marginal auricular vein (22 gauge, B. Braun, Germany) by injecting into it fentanyl (4 μg kg^− 1^, Rotexmedica GmbH, Germany), propofol (4 mg kg^− 1^, Fresenius Kabi GmbH, Germany) and atracurium (0.5 mg kg^− 1^, Hikma Pharma GmbH, Germany). Then, a secure airway was established using a standard endotracheal tube (ID 6.0–7.0 mm, Teleflex Medical, Ireland) under direct laryngoscopy. During the entire experiment, anaesthesia was maintained via continuous infusion of propofol (5–10 mg kg^− 1^ h^− 1^) and fentanyl (8–12 μg kg^− 1^ h^− 1^) as well as a balanced electrolyte infusion of 5 ml kg^− 1^ h^− 1^ (Sterofundin ISO, B. Braun, Germany). Volume-controlled ventilation was provided and monitored using an intensive-care respirator (Engstroem Care Station, GE Healthcare, Germany) with tidal volumes (V_t_) of 6–7 mL kg^− 1^, peak inspiratory pressures of 40 cmH_2_O, positive end-expiratory pressure of 5 cmH_2_O and a respiratory rate adapted to end-expiratory carbon dioxide (CO_2_) levels below 6 kPa (45 mmHg), which usually resulted in 20–30 breaths minute^− 1^.

After being anaesthetised, the animals were instrumented under the guidance of ultrasound with a central-venous catheter, pulse contour cardiac output system (PiCCO, Pulsion, Germany) and a Swan-Ganz catheter through introducer sheaths in the femoral veins and arteries as described before [13]. When the instrumentation was completed, the animals were screened again for any previously inapparent cardiopulmonary pathologies during base line measurements (i.e. ventricular defects or severe oxygenation impairments due to infections). Any afflicted animals would have been excluded from the trial and euthanised.

Following the health assessment, an oscillation catheter (Osypka Medical GmbH, Rheinfelden-Herten, Germany) was placed intravenously. The fasting animals were given an initial fluid bolus of 30 ml/kg warm balanced electrolyte solution and left to stabilise for 30 min before base line measurements were taken.

### Intervention

Following base line measurements, ventricular fibrillation was induced via the oscillation catheter (13.8 V current according to manufacturer’s recommendation) and the ventilator was disconnected. Monitor-confirmed cardiac arrest was permitted for eight minutes, and the animals were randomised into two groups by blinded drawing of 1 of 18 envelopes containing the respective chest compression device (9 animals per group):

- mCCD 1 - Continuous automated chest compressions via the LUCAS™ 2 device (Stryker® Corporation, Kalamazoo MI, USA) with a fixed rate of 100 min^− 1^ as described before [14].
- mCCD 2 - Continuous automated chest compressions via the Corpuls™ cpr device, using a Recboard and a standard size stamp (GS Elektromedizinische Geraete, Kaufering, Germany) with a fixed rate of 100 min^− 1^ and a set compression depth of 5 cm and positioning as described before [12].

During chest compressions, both groups were ventilated with a guideline-based ventilation regimen (V_t_ 8–10 ml kg^− 1^, F_i_O_2_ 1,0, RR 10 min^− 1^). After 8 min of continuous CPR, resuscitation measures were continued according to the advanced life support algorithm: 2 min compression cycles, rhythm analysis, defibrillation (200 J, bi-phasic), epinephrine (1 mg) and vasopressine (0.1 U kg^− 1^) administration as well as amiodarone (5 mg kg^− 1^) after the third and the sixth cycle. If ROSC was not achieved after the 10th cycle, the experiment was terminated. Animals achieving ROSC were switched back to standard ventilation and monitored for 8 h. During the monitoring period, mean arterial blood pressure was kept over 60 mmHg using a norepinephrine drip if necessary. The experiment was terminated with the animal being euthanised using high doses of propofol (200 mg) and potassium chloride (40 mmol).

### Measurements/sample collection

Cardiopulmonary data were constantly measured and collected during the duration of the experiment using a Datex Ohmeda S5 monitor (GE Healthcare, Munich, Germany). These include respiratory rate, ventilation pressures, oxygen fractions, oxygen saturation, intra-arterial blood pressure, pulmonary artery pressure, heart rate and core temperature. Additionally, blood gas analyses were performed at base line (“BLH”) 5 min into chest compressions (“BLS”), after the fourth shock (“ALS 1”) and after the eighth shock (“ALS 2”).

Ventilation/perfusion (V_A_/Q) analyses were performed at base line and during CPR (at “BLS”) using the micropore membrane inlet mass spectrometry facilitated multiple inert gas elimination technique (MMIMS-MIGET, Oscillogy LLC, Philadelphia, USA) as described before [13]. In short, subclinical, non-toxic doses of a saline solution containing six chemically inert gases with different elimination constants (sulphur hexafluoride, krypton, desflurane, enflurane, diethyl ether and acetone) were infused starting 20 min prior to measurements in order to reach an in vivo steady state. Blood samples from the pulmonary and femoral artery were taken and analysed via a mass spectrometer determining gas elimination during lung passage, thus allowing accurate V_A_/Q fraction determination for high, normal and low perfusion ratios as well as shunt volumes.

After termination, the thorax was examined using para- and substernal incisions and careful preparation in order not to inflict additional damage. Pericardium, pleura, rib cage and lung tissue were assessed using a scoring system developed by our group, consisting of 7 damage aspects (haematothorax, pneumothorax, rib fractures, sternal fractures, pericardial effusion/tamponade, blood in the gastric tube and blood in the tracheal tube, see Fig. 5). To support the clinical findings, sonographic analysis of the thorax was performed before the first incision, screening for pneumothorax or pleural effusion using a mobile device with a linear probe (Sonosite M, FUJIFILM Sonosite GmbH, Frankfurt, Germany). Exemplary pictures and videos as well as pictures of lung sonography and thoracic injuries are provided in the online supplement of this article.

Due to the pilot character of the study and the lack of any comparable data, no power calculation was performed and empirically reasonable group numbers were chosen. Statistical analyses were performed using 2-way ANOVA inter-group tests for repeated measurements as well as students-t-tests for single measurements via GraphPad Prism 8 software (GraphPad Software Inc., La Jolla, CA, USA). Data in the text are presented as mean (standard deviation). *p*-values < 0.05 were considered significant.

## Results

**Fig. 1:**
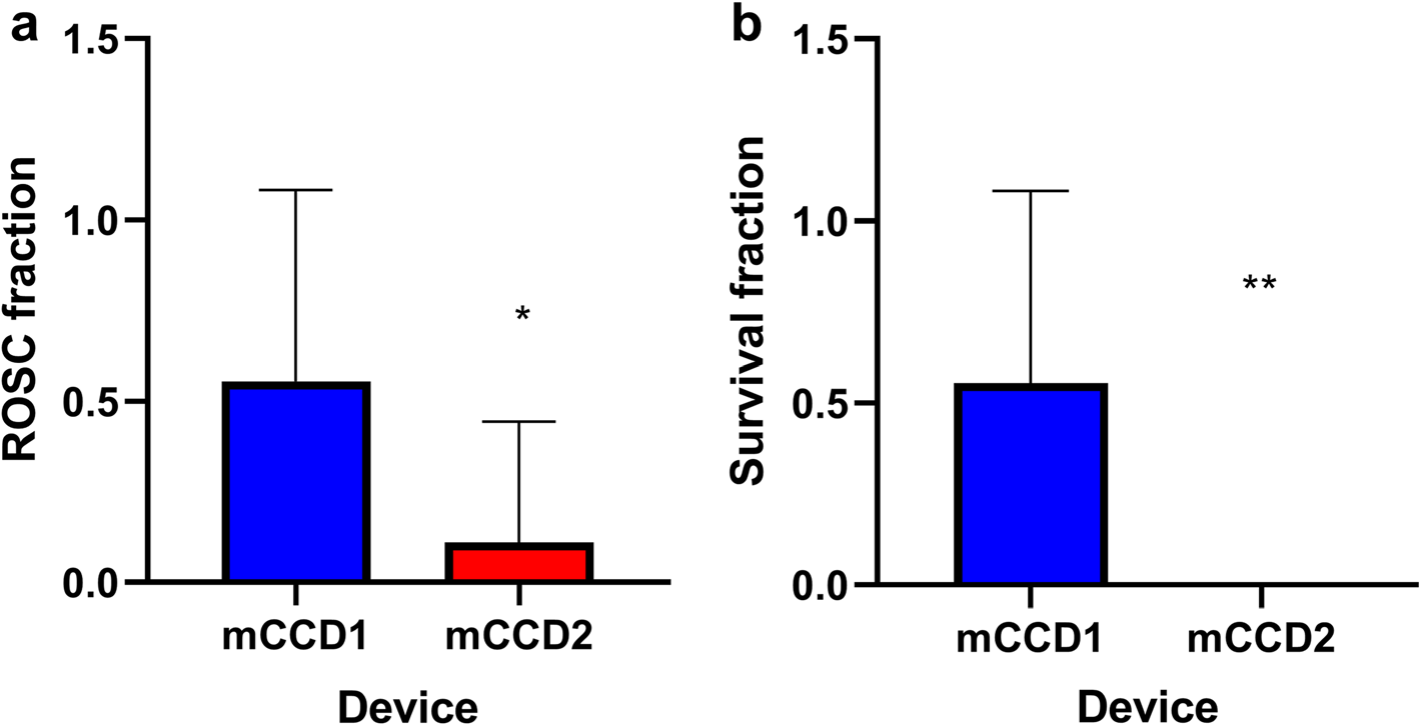
ROSC rate (**a**) and 8 h survival (**b**) of animals. The animal, that achieved ROSC in the mCCD2 group died before the monitoring period ended, resulting in ROSC rates of 55% in the mCCD1 group and 11% in the mCCD2 group (* = *p* < 0.05) and survival rates of 55% in the mCCD1 group and 0% in the mCCD2 group, respectively (** = *p* < 0.001)

**Fig. 2:**
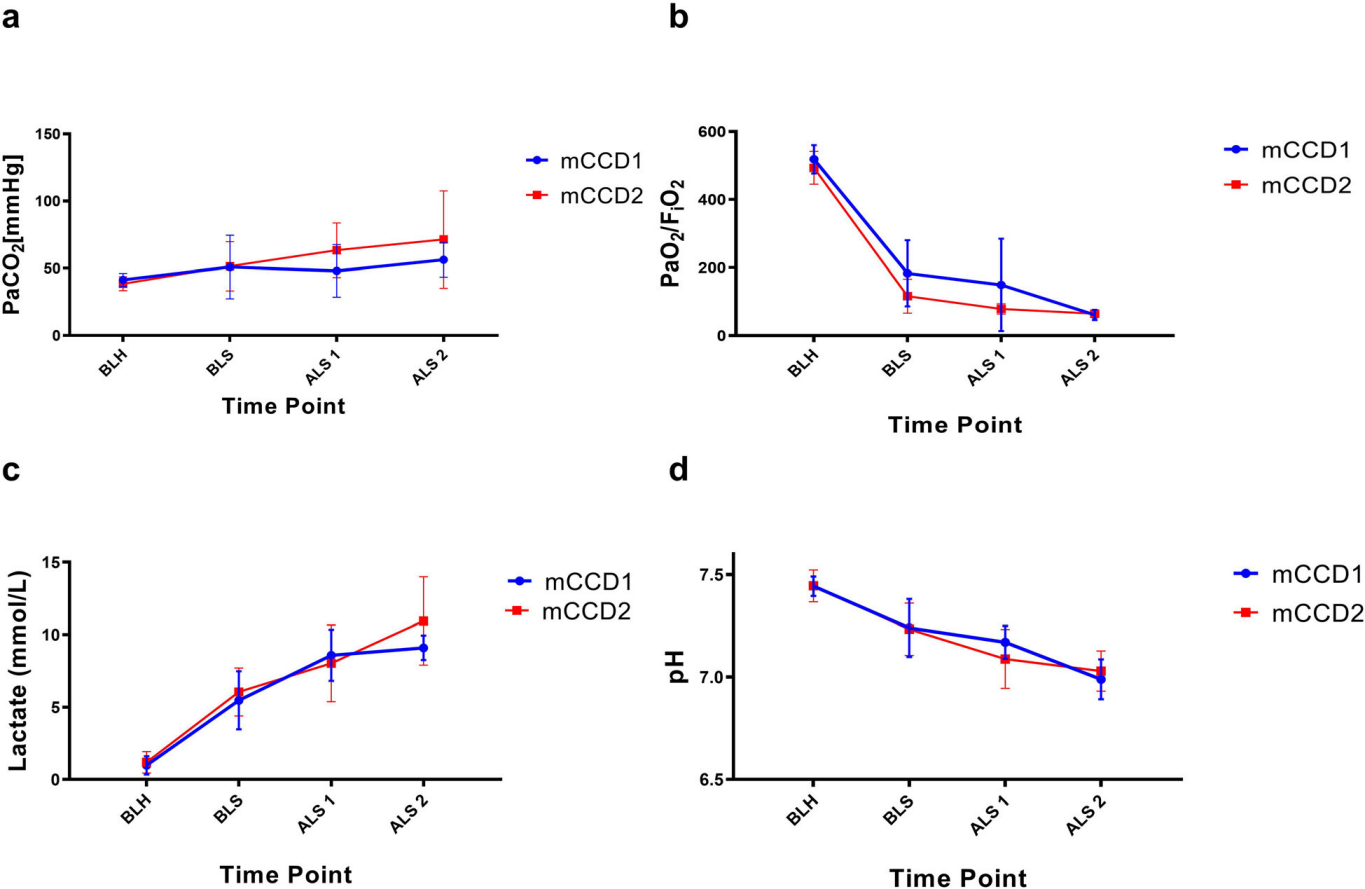
Arterial blood gas analyses at baseline (BLH), 5 min into basic life support (BLS), after four cycles (ALS 1) and eight cycles (ALS 2) of advanced life support. Neither decarboxylation (**a**) nor oxygenation (**b**) showed any differences between chest compression devices. Lactate generation (**c**) and pH (**d**) did not differ between groups

**Fig. 3:**
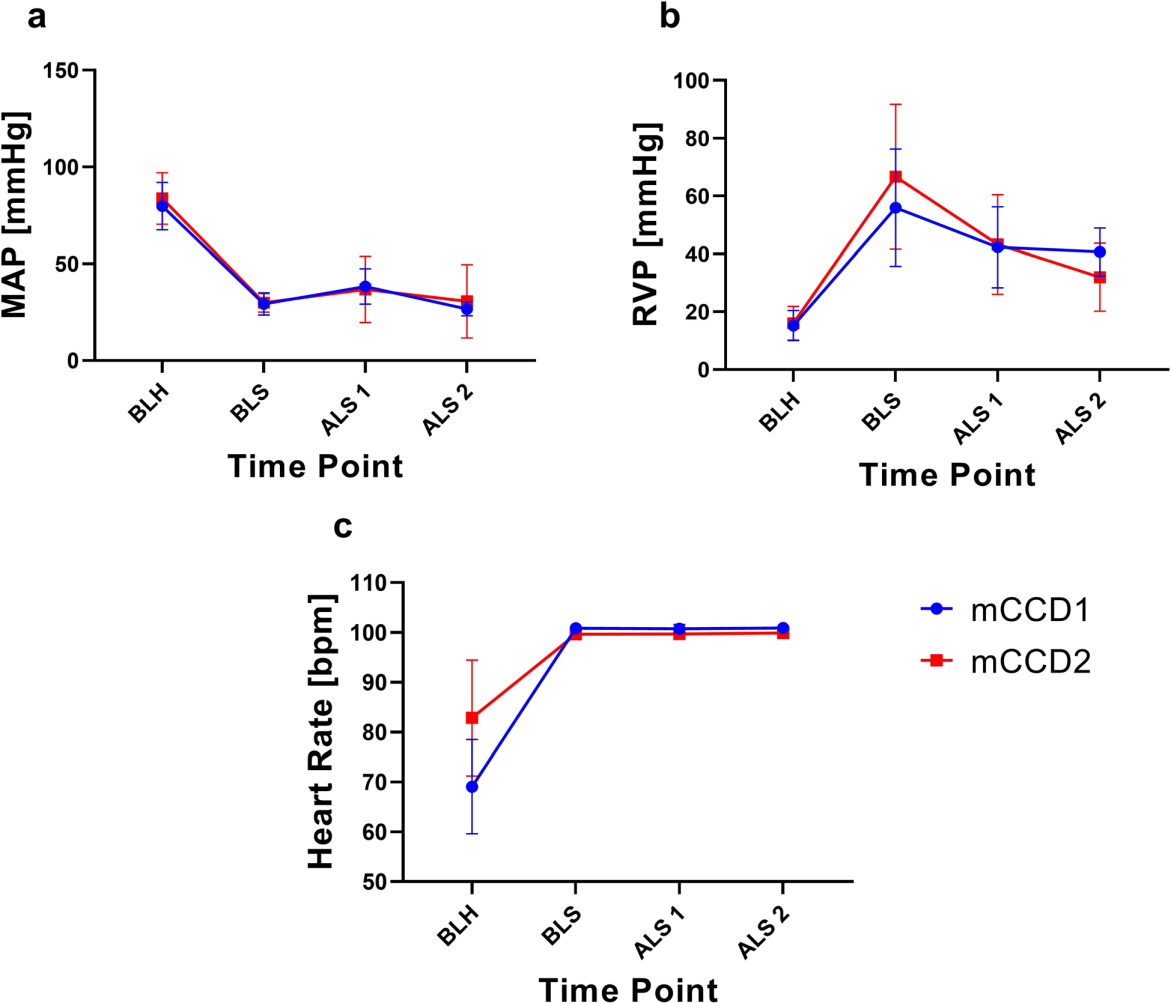
Haemodynamic parameters. Mean arterial pressure (MAP, **a**) and right ventricular pressure (RVP, **b**) showed no significant differences between groups. The generated heart rate (**c**) was adequate and stable in both groups. ALS 2 measurements include less values (4 in the mCCD1, 8 in the mCCD2 group) since all animals that achieved ROSC did so before the eighth cycle

A total of 18 experiments were performed. ROSC was achieved in five of the mCCD1 and one of the mCCD2 animals (*p* = 0.048). However, the mCCD2 animal died after 5 h showing tension pneumothorax, reducing the survival rate to 0 % and 55% in the mCCD1 group (*p* = 0.006) (Fig. 1). Blood gas analyses showed no statistically significant differences prior to cardiac arrest or during resuscitation (Fig. 2). Arterial and ventricular pressures did not differ between devices [mean arterial pressure mCCD1 vs. mCCD2: at BLH 79.8 (12.2) versus 83.8 (13.3); at BLS 29.4 (5.8) versus 29.9 (4.8); at ALS 1 38.3 (9.1) versus 36.9 (17.1); at ALS 2 26.7 (3.5) versus 30.7 (18.9) [all mmHg]] and the produced heart rate was sufficiently stable (Fig. 3).

**Fig. 4:**
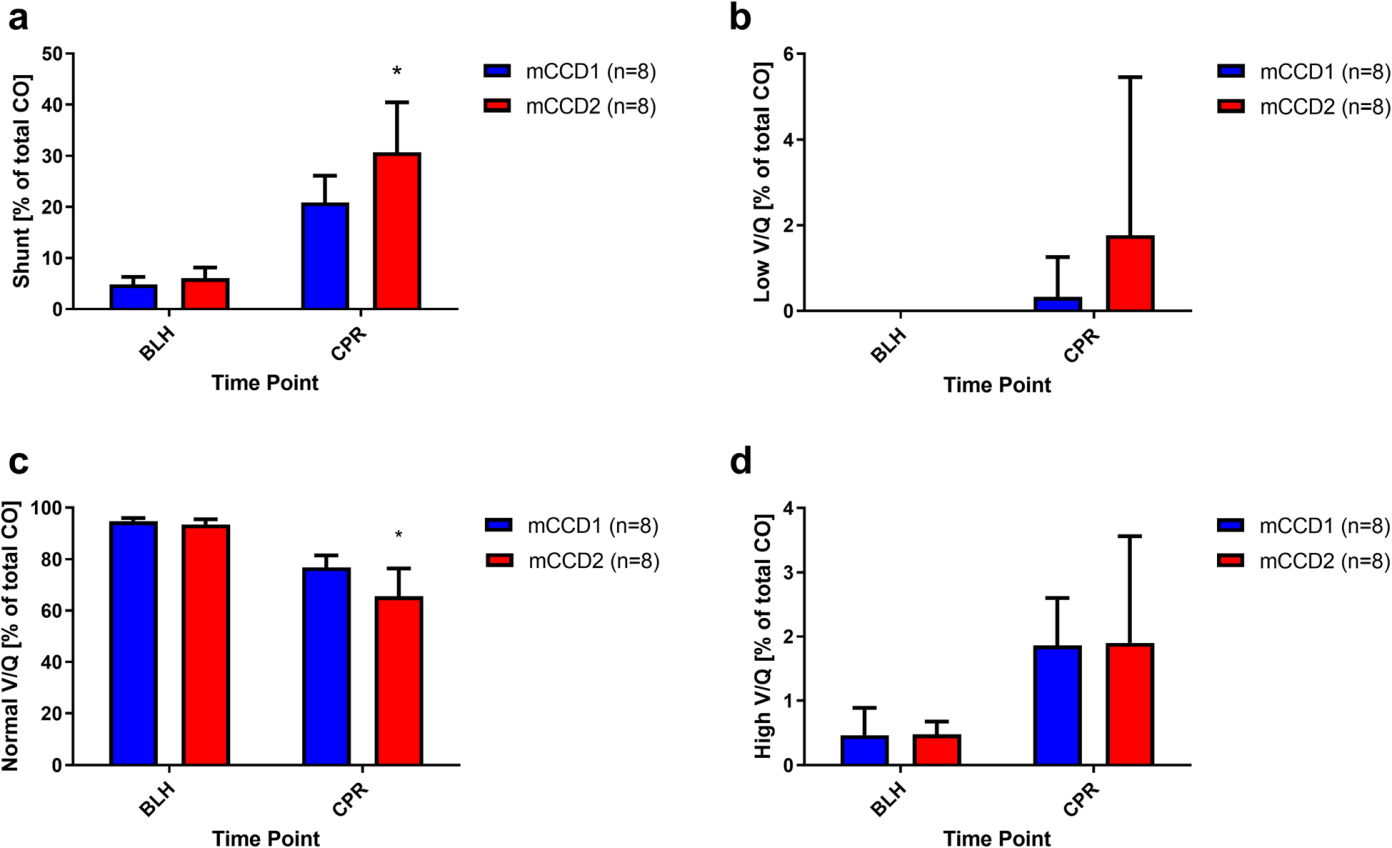
MMIMS-MIGET measurements. Relative shunt volumes expressed in per cent of cardiac output (CO) as an indication for a ventilation/perfusion mismatch and circulatory bypass of pulmonary gas exchange (**a**) Ratio of low (**b**), normal (**c**) and high (**d**) V/Q units (% of CO) symbolising hyper-(hypo)ventilated lung areas with insufficient perfusion to effectively contribute to gas exchange. Animals in the mCCD2 group showed significantly more shunt (*p* = 0.025) and less normal V/Q (*p* = 0.017) during CPR. Differences in high and low V/Q were not statistically significant. Analysed animal numbers are reduced due to technical difficulties during the trial, effectively disabling the MIGET device for 2 experiments. (* = *p* < 0.05)

Ventilation/perfusion analyses showed a significantly lower amount of bypassed lung areas (shunt) and a significantly higher normal V/Q fraction in mCCD1 animals during CPR [Shunt: 20.9 (5.2) versus 30.7 (9.7), *p* = 0.025; normal V/Q: 76.9 (4.7) versus 65.6 (10.8), *p* = 0.017 [all % of cardiac output]] (Fig. 4). Low and high V/Q fractions showed no significant differences. One animal per group could not be tested due to technical difficulties, bringing the analysed animals to 8 per group for the MIGET measurements.

**Fig. 5:**
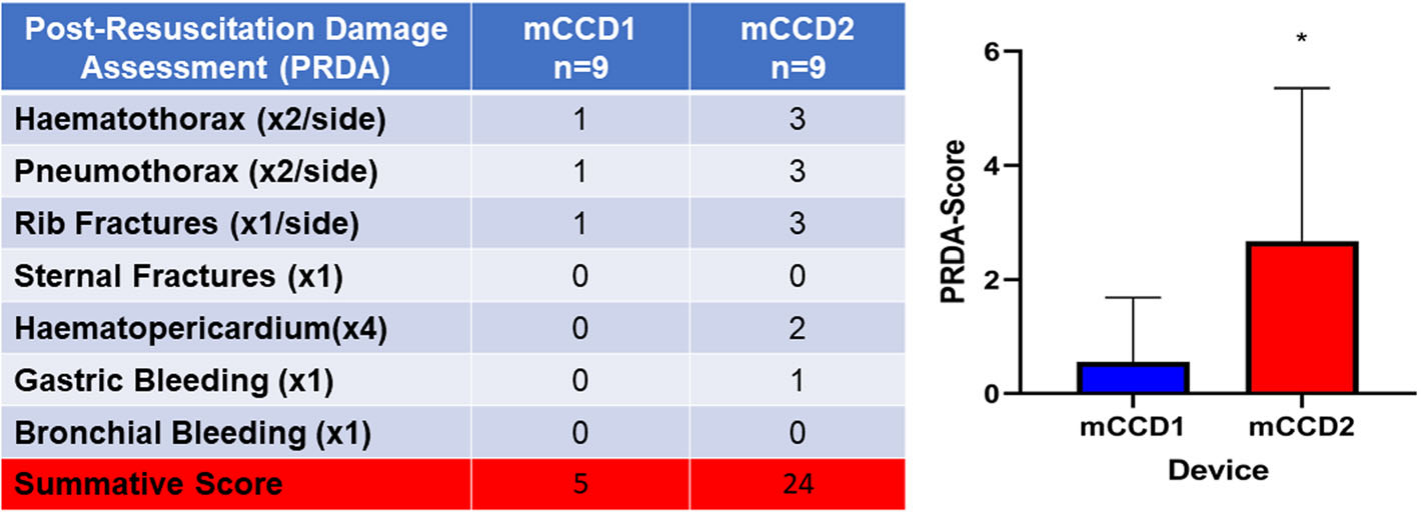
Proposal of a Post-Resuscitation Damage Assessment (PRDA) Score. The weighed scorecard (left) enables an objective and comparable damage assessment while simultaneously emphasising the clinical relevance of various injury patterns regularly seen after CPR, mechanical or otherwise. Statistical analysis of the thoracic injuries (right) shows significantly more severe damages after mCCD2 treatment [0.56 (1.1) versus 2.67 (2.7), *p* = 0.046), possibly explaining the poor ROSC rates. (* = *p* < 0.05)

Thoracic damage assessment showed substantial injuries in 2 of the mCCD1 and 6 of the mCCD2 animals (*p* = 0.06) with a substantially higher damage score after mCCD2 resuscitation (Fig. 5).

## Discussion

This study provides a randomised, controlled and detailed assessment of the impact of different mCCDs for experimental CPR in juvenile pigs. The presented data show a significantly higher likelihood of ROSC and survival, when chest compressions were performed using mCCD1. Additionally, we found less pulmonary shunt and increased normal ventilation/perfusion ratios during CPR, suggesting more efficient organ function. Generated arterial blood pressures were comparable during CPR while gas exchange showed no significant differences between treatment groups. Post-mortem thoracic damage assessment showed a tendency to more injuries caused by mCCD2. In-depth analysis of injury patterns using a self-developed, severity-weighed post-resuscitation damage assessment score showed significantly more severe and potentially life-threatening pathologies and a higher risk for intrathoracic bleeding with mCCD2, highlighting potential hazards of the device.

Mechanical chest compression devices have been shown before to cause more thoracic damages, especially when used over a longer period of time [15, 16] and compared to manual chest compressions [17, 18]. These usually range from lung contusions [19] over sternal and rib fractures [20] to haematothorax and haematopericardium [21], the latter being especially dangerous and immediately life-threatening independent of the original cause of cardiac arrest. However, the use of mCCDs can help to provide sufficient organ perfusion and adequate and stable compression depths in challenging scenarios like prolonged transport or difficult terrain [3, 22, 23]. The mCCD1 especially has been proven to not be inferior to standard treatment in terms of patient outcome and showed advantages in thoracic decompression as well [1], thus leading to its explicit mentioning in the ERC guidelines [8]. MCCD2 has been introduced for medical use in 2015 and is the only mCCD approved for the use on children 8 years and older, most likely due to its adjustable compression depth range between 2 to 6 cm and different compression stamp sizes. While the device showed significantly higher generated blood pressure [12] and higher mechanical pressures applied to the thoracic wall compared to mCCD1 in a porcine trial [10, 11], the already small experimental groups of 5 animals per device had been further reduced by two unplanned animal deaths before the start of the experiment and one device failure [24], which highlights the need of further assessment. No clinical data on human treatment or assessment on mCCD2, neither randomised nor observational, was publicly available at the time this trial was conducted except one yet unpublished observational study, which was initiated in 2017 [25].

Our study shows a higher probability of thoracic injury when mCCD2 was used. While this could be due to positioning and handling the device, the compression arm was placed and handled as recommended and compression was focused on the lower third of the sternum as described before [12]. It could be argued, that due to the fact that Corpuls animals did achieve ROSC only once, they were exposed to substantially longer resuscitation periods with a known risk for higher damages over time [7, 21]. While this is technically true with all animals having achieved ROSC before the sixth resuscitation cycle leading to eight to ten minutes less mCCD treatment, one of the two injured mCCD1 animals achieved ROSC and survived, leaving 3 animals without any injuries over the whole resuscitation period. However, the severity of the thoracic injuries found in mCCD2 animals suggested a direct correlation between damage and probability of ROSC. Additionally, the compromised pulmonary function depicted by MIGET measurements even before the first defibrillation already hinted at possible thoracic damage or at least insufficient perfusion, although this was neither confirmed by substantial differences in mean arterial blood pressures nor oxygenation impairments between the groups. While MIGET measurements are sophisticated, our group has shown their validity during CPR before [13]. Yet, since no other experimental group uses the technique in this context, systematic flaws cannot fully be excluded.

Discussing and comparing ROSC rates in experimental protocols of large animals is difficult. Depending on no-flow time, time to drug administration and defibrillation methods as well as ventilation settings, ROSC rates ranging from 30% to over 80% are regularly reported [26, 27]. While differences between groups can occur randomly and might be due to the small sample sizes [13], they usually do not reach statistical significance. Additionally, as in this manuscript, power analyses are often not performed due to the fact that insufficient preliminary data on the subject exist to reasonably calculate and standard group sizes of 7 to 10 animals have been established. However, the fact that not one animal survived after mCCD2 treatment combined with the damage severity confirmed in those animals causes our group to be confident that those results are sound. One explanation could be the aforementioned higher mechanical force applied during mCCD2 chest compression, with a peak of over 500 Nm compared to about 350 Nm with mCCD1 [10]. This could be due to the differently designed stamps with the mCCD1 providing a stamp with a diameter of 6 cm and an additional 13 cm diameter rubber suction cup and mCCD2 providing an adult stamp with 8 cm diameter without rubber lining. The possibility of a defective device can also not be fully excluded, although we did not encounter any technical problems during the experiments and both machines worked without any obvious errors. Another reason could be the automated compression depth compensation mechanism unique to mCCD2. While we were not able to reliably measure actual chest compression depths in this study due to technical limitations, the different anatomy of the porcine thorax with a larger diameter [28] and tendency to an almost keel breast-like appearance in piglets could have triggered an overcompensation of the device, causing more forceful compressions and, subsequently, more organ damage, thus explaining the deleterious outcomes. Although the animals used in this study are smaller in size compared to the human patients the mCCDs are designed for, the specific thoracic anatomy demands an adjusted compression depth not linearly correlated to body weight to achieve adequate pressures. Piglets of the same age and weight have been shown to be an adequate surrogate and were used in the previous mCCD2 evaluation as well [12], thus providing sufficient comparability.

A standardised assessment of thoracic damages after CPR could be helpful to stratify and compare patient collectives and eventually identify relevant prognostic effects of certain injuries. However, to the best of our knowledge, no reliable scoring system or comparable assessment tool has been published or evaluated so far. While forensic analyses on patients after CPR have been conducted, they tend to focus on bone fractures [15, 16] or lung contusions alone [19], rarely stating epicardial bleeding or haematothoraces [21, 29, 30]. Additionally, all of those studies rely on retrospective data and do not correlate injuries to patient outcomes. For this trial, we tried to establish a standardised assessment tool, which is supposed to help compare post-resuscitation damage patterns and facilitate their adequate statistical analysis. To achieve this, we used the most common injuries as seen in the cited forensic studies and weighed them according to their immediate clinical danger to the patient. Since this is highly subjective and not based on confirmed statistical data, this scorecard still has to be adequately validated on patient data and, at best, correlated to eventual outcomes to add a predictive value. Nevertheless, the application of this damage assessment tool helped to better evaluate the damage patterns we identified during this trial and could be helpful in future experiments of other groups.

## Conclusion

The LUCAS 2 device shows superior resuscitation outcomes and less thoracic injuries compared to Corpuls cpr when used for experimental CPR in juvenile pigs. Researchers should be aware that different mCCDs for experimental studies may significantly influence the respective outcome of resuscitation studies and affect comparability of different trials. Controlled human and animal CPR studies and a standardised post-resuscitation injury evaluation could help to confirm potential hazards but more data would be needed to validate the suggested score for actual clinical use.

## Supplementary Information

**Additional file 1: Figure S1.** Damage patterns after mCCD2 resuscitation. Haematothorax in M and B-mode (**left and middle**). Sonography was performed in a mid-axillar line between the 6th and the seventh rib. After opening the thorax, massive haemothorax and haematopericardium was discovered (**right**). **Figure S2.** Exemplary pictures of post-mortem lung tissue. Ventral and dorsal view of the lung of an mCCD2 animal (**left**) and dorsal and ventral views after mCCD1 resuscitation (**right**). Extensive atelectasis is seen in both groups after resuscitation. Although the presented pictures look different, no statistically significant differences in direct pulmonary damage (bleeding, rupture, atelectasis, bullae) could be found.

## Data Availability

All data generated or analysed during this study are included in this published article [and its supplementary information files].

## Abbreviations

CPR: Cardiopulmonary resuscitation
ERC: European Resuscitation Council
mCCD: Mechanical chest compression device
MIGET: Multiple inert gas elimination technique
ROSC: Return of spontaneous circulation
V/Q: Ventilation/Perfusion ratio
