# Supplementary material for: Standardized post-resuscitation damage assessment of two mechanical chest compression devices: a prospective randomized large animal trial
Source: Scand J Trauma Resusc Emerg Med. 2021 Jun 5;29:79. doi: 10.1186/s13049-021-00892-4 (PMC8179713; doi:10.1186/s13049-021-00892-4)
Supplement: Supplementary file 1 — Additional file 1: Figure S1. Damage patterns after mCCD2 resuscitation. Haematothorax in M and B-mode (left and middle). Sonography was performed in a mid-axillar line between the 6th and the seventh rib. After opening the thorax, massive haemothorax and haematopericardium was discovered (right). Figure S2. Exemplary pictures of post-mortem lung tissue. Ventral and dorsal view of the lung of an mCCD2 animal (left) and dorsal and ventral views after mCCD1 resuscitation (right). Extensive atelectasis is seen in both groups after resuscitation. Although the presented pictures look different, no statistically significant differences in direct pulmonary damage (bleeding, rupture, atelectasis, bullae) could be found. [file 13049_2021_892_MOESM1_ESM.docx]

Supplement to “**Standardized Post-Resuscitation Damage Assessment of Two Mechanical Chest Compression Devices: A Prospective Randomized Large Animal Trial”**

By R. Ruemmler et al.


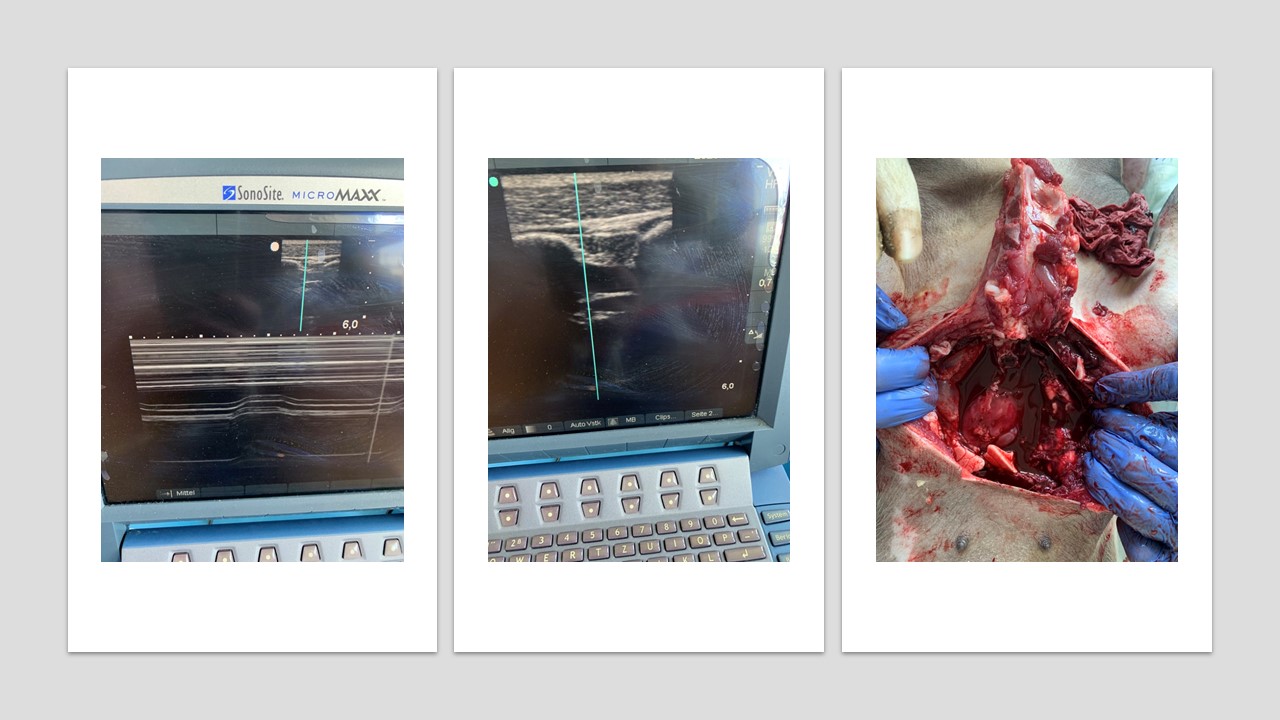


**Figure S1**: Damage patterns after mCCD2 resuscitation. Haematothorax in M and B-mode (**left and middle**). Sonography was performed in a mid-axillar line between the 6^th^ and the seventh rib. After opening the thorax, massive haemothorax and haematopericardium was discovered (**right**).


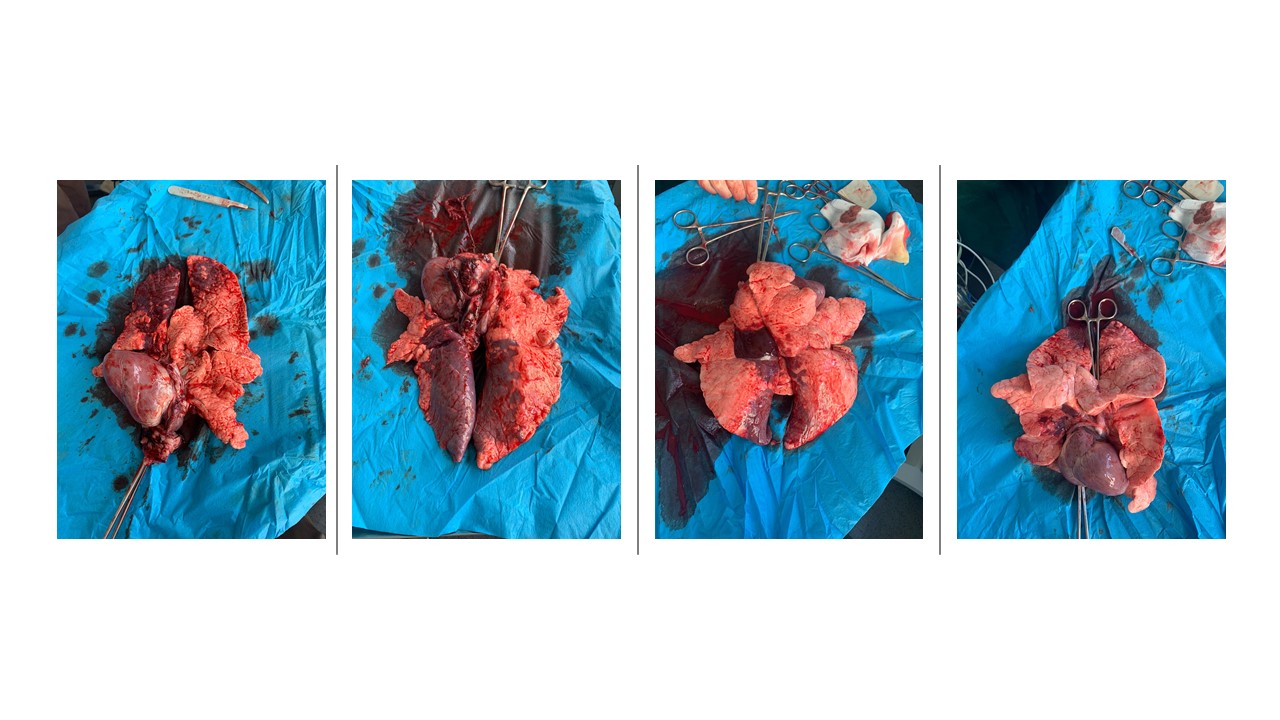


**Figure S2:** Exemplary pictures of post-mortem lung tissue. Ventral and dorsal view of the lung of an mCCD2 animal (**left**) and dorsal and ventral views after mCCD1 resuscitation (**right**). Extensive atelectasis is seen in both groups after resuscitation. Although the presented pictures look different, no statistically significant differences in direct pulmonary damage (bleeding, rupture, atelectasis, bullae) could be found.
